# Implementation of Non-Pharmaceutical Interventions by New York City Public Schools to Prevent 2009 Influenza A

**DOI:** 10.1371/journal.pone.0050916

**Published:** 2013-01-15

**Authors:** Simon G. Agolory, Oxiris Barbot, Francisco Averhoff, Don Weiss, Elisha Wilson, Joseph Egger, Jeffery Miller, Ikechukwu Ogbuanu, Sabrina Walton, Emily Kahn

**Affiliations:** 1 Epidemic Intelligence Services, Centers for Disease Control and Prevention, Atlanta, Georgia, United States of America; 2 Department of Health, City of Baltimore, Baltimore, Maryland, United States of America; 3 Division of Global Migration and Quarantine, Centers for Disease Control and Prevention, Atlanta, Georgia, United States of America; 4 Department of Health and Mental Hygiene, City of New York, New York, United States of America; 5 SciMetrika LLC, Research Triangle Park, North Carolina, United States of America; The University of Hong Kong, Hong Kong

## Abstract

**Introduction:**

Children are important transmitters of influenza in the community and a number of non-pharmaceutical interventions (NPIs), including hand washing and use of hand sanitizer, have been recommended to mitigate the transmission of influenza, but limited information is available regarding schools' ability to implement these NPIs during an influenza outbreak. We evaluated implementation of NPIs during fall 2009 in response to H1N1 pandemic influenza (pH1N1) by New York City (NYC) public schools.

**Methods:**

From January 25 through February 9, 2010, an online survey was sent to all the 1,632 NYC public schools and principals were asked to participate in the survey or to designate a school nurse or other school official with knowledge of school policies and characteristics to do so.

**Results:**

Of 1,633 schools, 376(23%) accessed and completed the survey. Nearly all respondents (99%) implemented at least two NPIs. Schools that had a Flu Response Team (FRT) as a part of school emergency preparedness plan were more likely to implement the NPI guidelines recommended by NYC public health officials than schools that did not have a FRT. Designation of a room for isolating ill students, for example, was more common in schools with a FRT (72%) than those without (53%) (p<0.001).

**Conclusions:**

Implementing an NPI program in a large school system to mitigate the effects of an influenza outbreak is feasible, but there is potential need for additional resources in some schools to increase capacity and adherence to all recommendations. Public health influenza-preparedness plans should include school preparedness planning and FRTs.

## Introduction

As with previous influenza outbreaks in which school-aged children were disproportionately affected [1]–[6], the 2009 pandemic influenza A virus (pH1N1) outbreak predominantly affected school-aged children [7]–[12]. The pH1N1outbreak was more pronounced among children and was associated with high rates of pediatric deaths and hospitalizations compared with previous influenza seasons [13]. In New York City (NYC), the first case of pH1N1 was confirmed in a high school student on April 23, 2009, a week after the first cases in the United States were diagnosed in two children in California [14]–[16]. Before the first NYC case was confirmed, 33% of students from one NYC school were found to have symptoms consistent with influenza infection, according to a survey conducted by NYC health officials [17], [18].

Concerns about the spread of pH1N1 in NYC prompted the NYC Department of Health and Mental Hygiene (DOHMH) to develop guidelines to mitigate the spread of the virus in schools [19]. The guidelines were based on guidance from the Centers for Disease Control and Prevention (CDC) on the use of non-pharmaceutical interventions (NPIs) in schools [20]. Several published studies suggest that using NPIs can reduce spread of respiratory infections, including influenza, in schools and other settings [21]–[25]. CDC-recommended NPIs include hand washing and/or use of hand sanitizer, immediate isolation of students with influenza-like illness (ILI), routine cleaning of surfaces that students and staff touch frequently, teaching proper hand washing and respiratory etiquette in schools, and the use of face masks by persons exposed to patients with suspected influenza [20].

The DOHMH and the Department of Education (DOE), through the Office of School Health (OSH), shared responsibility for implementing the guidelines. The OSH, a jointly administered program between the DOE and DOHMH, provides health care and preventive services at NYC public schools. OSH assisted schools in implementing the DOHMH guidelines for preventing the 2009 spread of pH1N1during the outbreak. In addition to the CDC recommendations, the DOHMH guidelines recommended that each NYC school form a Flu Response Team (FRT) made up of school administrators, teachers, parents, and school-based health care workers to oversee implementation of the DOHMH guidelines.

To evaluate the capacity of schools to implement and adhere to an extensive NPI program, the DOE, DOHMH, and CDC conducted a web-based survey of NYC public schools in all the five boroughs (Brooklyn, Bronx, Manhattan, Queens and Staten Island) during January 2010, after the peak of the pH1N1 pandemic in the United States [19], [20].

## Methods

### Human Subjects Approval Statement

CDC and NYC DOHMH Human Subjects Coordinators reviewed the study protocol, the questionnaire, and all the other study materials and determined that the study did not constitute human subjects research.

### Procedures

A web-based survey of 1,632 NYC public schools was conducted from January 25 through February 9, 2010. In a joint e-mail invitation from the DOE and DOHMH, principals of all NYC public schools were asked to participate in the survey or to designate a school nurse or other school official with knowledge of school policies and characteristics to do so. The e-mail, containing a web address for a secure online survey instrument, was sent to schools by DOE, using a general electronic mailing list. DOE and DOHMH sent reminder e-mails to principals and school nurses during the study period encouraging their participation.

We obtained data from the NYC Comprehensive Education Plan (CEP) school Demographics and Accountability Snapshot database [26] regarding the characteristics of surveyed schools, including instructional level (primary, middle, high school, other), borough, poverty rate (The percentage of public schools where more than three-quarters of students are eligible for free or reduced price lunch), school size, student ethnicity, and receipt of federal Title 1 funds (a federal program that provides financial assistance to Local Education Agencies and schools with high numbers or high percentages of poor children) [27].

### Questionnaire

The questionnaire requested information about planning, educational messages, communication strategies, handling sick students, hand hygiene, and respiratory etiquette during the spring term of 2008–09 and the fall term of 2009–10 academic years.

### Data Analysis

Our analysis was limited to public schools that could be matched to the CEP database for the 2008–09 school year. Because the grade level structure was not uniform across the school system, we divided schools into the following categories: Kindergarten (K) -5^th^ grade; K-8^th^ grade, K-12^th^ grade; 6^th^–8^th^ grade; 6^th^–12 grade; and 9^th^–12^th^ grade. Additionally, we categorized schools according to whether there was a school wide title I program [27] and by poverty rate in the school into the following groups: 0–24.9%; 25–49.9%, 50–74.9%, and 75–100%. Schools were categorized into three groups by enrollment size: <500, 500–1000, and >1000 students. We used the Satterthwaite t-test to determine whether responding and non responding schools differed in the distribution of students by race and ethnicity and the chi-square test and Fisher's exact test for trend to assess whether the two groups of schools differed by CEP characteristics. Within the sample of responding schools, these tests were also used to determine whether schools with an FRT and schools reporting elevated ILI in the spring of 2009 differed according to school characteristics. Missing values were excluded from statistical analyses; percents reflect the number of respondents who gave similar responses to a question out of all the respondents who answered that question. For all tests of statistical association, we used two-sided probabilities with an α of 0.05. Data were analyzed by using SAS 9.3 (SAS Inc, Cary, NC, USA).

## Results

Of the 1,632 general education public schools in NYC, only 376 (23%) accessed the online- survey. Schools' demographic data from CEP were available for 93% (1,518/1,632) of all schools, including all respondents. Non-respondents were less likely than respondents to have 9–12^th^ grades (p<0.001) and to have black or African American (p = 0.012) or Hispanic/Latino students (p<0.001). Responding and non-responding schools did not differ in poverty rate (p = 0.293) or Title I eligibility (p = 0.749) (Table 1).

**Table 1:** Characteristics of all NYC public schools by survey participation status, 2009–10 academic year.

Characteristic	No. (%)of NYC public schools	Survey participation		p-value^a^
		No. (%) of Respondents	No. (%) of Non-Respondents	
**Instructional level**	***n = 1,517***	***n = 376***	***n = 1,142***	***<0.001*** b
Elementary schools (K-5)	565 (37%)	190 (51%)	375 (33%)	
K-8 Schools	203 (13%)	75 (20%)	128 (11%)	
K-12 Schools	36 (2%)	8 (2%)	28 (2%)	
6–8 (Middle schools)	259 (17%)	42 (11%)	217 (19%)	
6–12 (Middle/High)	88 (6%)	12 (3%)	76 (7%)	
9–12 (High schools)	352 (23%)	37 (10%)	316 (28%)	
Other	14 (1%)	12 (3%)	2 (<1%)	
**School Borough**	***n = 1,517***	***n = 376***	***n = 1,141***	***<0.001*** b
Bronx	359 (24%)	49 (13%)	310 (27%)	
Brooklyn	481 (32%)	103 (27%)	378 (33%)	
Manhattan	294 (19%)	59 (16%)	235 (21%)	
Queens	315 (21%)	139 (37%)	176 (15%)	
Staten Island	64 (4%)	26 (7%)	42 (4%)	
**Title I eligibility** *				
**Title I**	***n = 1,497***	***n = 364***	***n = 1,133***	*0.749* b
School wide program	1293 (86%)	312 (86%)	981 (87%)	
No School wide program	204 (14%)	52(14%)	152 (13%)	
**Poverty rate** †	***n = 1,505***	***n = 364***	***n = 1,141***	*0.293* b
0–24.9%	153 (10%)	44 (12%)	109 (10%)	
25–49.9%	182 (12%)	46 (13%)	136 (12%)	
50–74.9%	547 (36%)	137 (38%)	410 (36%)	
75–100%	623 (41%)	137 (38%)	486 (43%)	
**School size**	***n = 1,505***	***n = 364***	***n = 1,141***	***<0.001*** b
<500 students	731 (49%)	132 (36%)	599 (53%)	
500–1000 students	560 (37%)	175 (48%)	385 (34%)	
>1000 students	214 (14%)	57 (16%)	157 (14%)	
**Ethnicity** ¶		***n = 376*** **%mean (95% CI)**	***n = 1,142*** **%mean (95% CI)**	***P-va1ue*** c
American Indian or Alaska Native		<1% (0.43–0.53)	<1% (0.46–0.52)	0.702
Black or African American		**32% (29.0–35.1)**	**36% (34.9–38.1)**	**0.012**
Hispanic or Latino		**36% (33.7–38.8)**	**42% (40.7–43.7)**	**<0.001**
Asian or Native Hawaiian/Other Pacific Islander		**15% (13.1–17.1)**	**9% (8.4–10.1)**	**<0.001**
White		**15% (13.3–17.6)**	**11% (9.9–12.0)**	**0.003**

a Participating schools compared to non-participating schools.

b Chi-square test.

c Satterthwaite T-test of Mean Difference, two-sided exact **Pr>|t|**.

* A federal program that provides financial assistance to Local Education Agencies and schools with high numbers or high percentages of poor children.

† The percentage of public schools where more than three quarters of students are eligible for free or reduced price lunch.

¶ Proportion of each ethnic group in schools.

### Planning

Sixty-nine percent (224/325) of respondents reported having an FRT in their school in which the principal (72%), school nurse (92%), and parent coordinator (64%) were highly involved. Schools reporting an FRT were more likely to have K-5 and K-8 grades compared with schools that did not (Table 2). Sixty-five percent (208/314) of respondents stated the school had a separate (holding) room used for isolating children with ILI symptoms, and 60% (124/314) of respondents reported that the holding room was the nurse's office. Respondents with an FRT in their school were more likely to report a holding room (72%, 157/314) compared with respondents without an FRT (46%, 36/314) (p<0.001) and were more likely to report isolating student and staff with ILI symptoms (p<0.001).

**Table 2:** Characteristics of NYC public schools responding to the web-based survey, according to whether the school had a Flu Response Team.

Characteristic	Survey participation			Exact test p-value^a^^,^ b
	No. (%) Respondent with Flu Response Team	No. (%) Respondent without Flu Response Team	% Don't Know	
**Instructional level**	***n = 224***	***n = 79***	***n = 22***	**0.018**
Elementary schools (K-5)	124 (54%)	40 (51%)	8 (36%)	
K-8 Schools	45 (20%)	14 (18%)	4 (18%)	
K-12 Schools	3 (1%)	5 (6%)	0	
6–8 (Middle schools)	27 (12%)	8 (10%)	2 (9%)	
6–12 (Middle/High)	6 (3%)	2 (3%)	0	
9–12 (High schools)	20 (9%)	5 (6%)	6 (27%)	
Other	3 (1%)	5 (6%)	2 (9%)	
**School Borough**	***n = 224***	***n = 79***	***n = 22***	**0.045**
Bronx	30 (13%)	8 (10%)	5 (23%)	
Brooklyn	56 (25%)	21 (27%)	7 (32%)	
Manhattan	33 (15%)	14 (18%)	7 (32%)	
Queens	84 (38%)	34 (43%)	3 (14%)	
Staten Island	21 (9%)	2 (3%)	0	
**Title I eligibility** *				
**Title I**	***n = 221***	***n = 74***	***n = 20***	0.097
School wide program	193 (87%)	61 (82%)	15 (75%)	
No school wide program	28 (13%)	13 (18%)	2 (10%)	
**Poverty rate** †	***n = 221***	***n = 74***	***n = 20***	0. 58
0–24.9%	27 (12%)	10 (14%)	3 (15%)	
25–49.9%	26 (12%)	10 (14%)	0	
50–74.9%	91 (41%)	24 (32%)	8 (40%)	
75–100%	77 (35%)	30 (41%)	9 (45%)	
**School size**	***n = 221***	***n = 74***	***n = 20***	<0.285
<500 students	73 (33%)	28 (38%)	10 (50%)	
500–1000 students	109 (49%)	39 (53%)	8 (40%)	
>1000 students	39 (18%)	7 (9%)	2 (10%)	
**Ethnicity** ¶	***n = 224*** **%mean (95% CI)**	***n = 79*** **%mean (95% CI)**	***n = 22*** **%mean (95% CI)**	
American Indian or Alaska Native	<1% (0.40–0.52)	1% (0.47–0.77)	<1% (0.25–0.62)	0.249§
Black or African American	30% (26.5–34.4)	39% (32.0–45.9)	34% (19.8–48.5)	0.197§
Hispanic or Latino	36% (32.9–39.5)	33% (27.3–37.7)	45% (31.7–58.3)	0.989§
Asian or Native Hawaiian/Other Pacific Islander	15% (12.5–17.7)	15% (10.6–20.0)	9% (1.9–16.0)	0.987§
White	17% (14.0–20.1)	12% (8.3–16.2)	11% (4.4–17.4)	0.062§
**Responses to pH1N1**				
Designated or had a room used exclusively for separating ill students (holding room) (n = 318)	**157/219 (72%)**	**41/78 (53%)**	**10/21 (48%)**	**0.001**
Educated or encouraged students to use respiratory etiquette (n = 321)	**220/221 (100%)**	**78/79 (99%)**	**19/21 (90%)**	**0.001**
Educated or encouraged students to use proper hand hygiene (n = 321)	**221/221 (100%)**	**78/79 (99%)**	**19/21 (90%)**	**0.002**
More than 75% of teachers taught curriculum on hand hygiene and respiratory etiquette (n = 320)				
during September 2009	**142/220 (65%)**	**35/79 (44%)**	**5/21 (24%)**	**<0.001**
During October 2009	**138/220 (63%)**	**30/79 (38%)**	**5/21 (24%)**	**<0.001**
During November 2009	**118/220 (54%)**	**28/79 (35%)**	**5/21 (24%)**	**<0.001**
During December 2009	**119/220 (54%)**	**27/79 (34%)**	**5/21 (24%)**	**<0.001**
Tissue was made available in these locations in school (n = 320)				
Classrooms	**197/220 (90%)**	**73/79 (92%)**	**15/21 (71%)**	**0.022**
Bathrooms	116/220 (53%)	40/79 (51%)	10/21 (48%)	0.876
Hallways	3/220 (1%)	3/79 (4%)	0	0.317
Admin offices	**196/220 (89%)**	**59/79 (75%)**	**16/21 (76%)**	**0.005**
Lunch room	70/220 (32%)	23/79 (29%)	6/21 (29%)	0.879
Hand sanitizer was made available in these locations in school (n = 320)				
Classrooms	**192/220 (87%)**	**70/79 (89%)**	**14/21 (67%)**	**0.025**
Bathrooms	82/220 (37%)	24/79 (30%)	4/21 (19%)	0.168
Hallways	22/220 (10%)	8/79 (10%)	0	0.313
Admin offices	**189/220 (86%)**	**59/79 (75%)**	**14/21 (67%)**	**0.015**
Lunchroom	**85/220 (39%)**	**30/79 (38%)**	**2/21 (10%)**	**0.029**
Cover your cough posters were made available in these locations in school (n = 320)				
Classrooms	102/220 (46%)	33/79 (42%)	6/21 (29%)	0.261
Bathrooms	98/220 (45%)	37/79 (47%)	9/21 (43%)	0.92
Hallways	**186/220 (85%)**	**49/79 (62%)**	**12/21 (57%)**	**<0.001**
Admin offices	105/220 (48%)	29/79 (37%)	10/21 (48%)	0.233
Lunchroom	136/220 (62%)	41/79 (52%)	10/21 (48%)	0.179
Frequently cleaned areas and items that are more likely to have frequent hand contact (n = 318)	**186/219 (85%)**	**58/78 (74%)**	**12/21 (57%)**	**0.016**
Communicated with students about flu at least once using the following methods (n = 317)				
Letters or handouts	**198/218 (72%)**	**61/78 (22%)**	**19/21 (5%)**	**<0.001**
School assemblies	**157/218 (72%)**	**41/78 (53%)**	**9/21 (43%)**	**0.001**
E-mails	**26/218 (12%)**	**9/78 (12%)**	**5/21 (24%)**	**0.001**
Social Media (Facebook, twitter, etc)	**3/218 (1%)**	**0**	**2/21 (10%)**	**<0.001**
World Wide Web	**83/218 (38%)**	**12/78 (15%)**	**5/21 (24%)**	**<0.001**

a Participating schools compared with nonparticipating schools.

b Chi-square test.

§ Satterthwaite T-test of Mean Difference, two-sided exact **Pr>|t|**.

* A federal program that provides financial assistance to Local Education Agencies and schools with high numbers or high percentages of children in families with household income below federal poverty levels/// (or something to define ‘poor’.

† The percentage of public schools where ≥75% of students are eligible for free or reduced price lunch.

¶ Proportion of each ethnic group in schools.

### Health Education

During the 2009 fall term, more than 99% (317/320) of respondents reported educating and/or encouraging students about proper hand hygiene and respiratory etiquette. When asked if a hand hygiene and respiratory etiquette curriculum was taught by teachers in their schools, 65% (182/280) reported that the topic was taught during September 2009, 62% (173/280) during October, 55% (151/273) during November, and 56% (151/269) during December. “Cover your cough” posters were reported to be available in school hallways by 77% (247/320) of respondents, in lunchrooms by 58% (187/320) of respondents, and in classrooms by 56% (179/320) of respondents. Respondents from schools with an FRT were more likely than respondents from schools without an FRT to report teaching curriculum on proper hand hygiene and respiratory etiquette during the study period (p<0.001) (Table 2).

### Communication

Monthly communication with students about pH1N1 during fall 2009 by using school-wide handouts was reported by 40% (120/301), weekly communication by 27% (81/301) of respondents, and 21% (64/301) of respondents reported communication with students through this method only once.. Use of student assemblies was reported by 75% (215/285) of respondents. Electronic communications were reported less frequently; only 2% (5/273) of schools ever used social media (Facebook, Twitter, etc), 15% (40/273) used e-mail, and 37% (100/267) used a website to communicate with students about pH1N1. Respondents from schools with an FRT were more likely to report communicating with students about influenza using letters, school assemblies, or other form of communication compared with respondents in schools without an FRT (all p-values <0.001) (Table 2). Schools reported communicating with parents mostly through weekly (33% [98/301]) or monthly (40% [119/301]) letters. With respect to electronic media for communication with parents about pH1N1, respondents reported that less than 2% (5/263) of schools used social media, 5% (13/266) used mass texting, 33% (86/258) used e-mail, and 51% (129/252) used the Internet. Influenza communications prepared in languages other than English were used by 92% (278/303) of responding schools.

### Sanitation and hygiene

During the fall 2009 term, tissue, hand sanitizer, and soap were reported available in at least one location in the school by nearly all respondents. Respondents stated that parents supplied tissues in 65% (205/317) and hand sanitizer in 53% (164/310) of schools, and individual faculty or staff supplied tissue in 60% (189/317) and hand sanitizer in 58% (179/310) of schools. Approximately 82% (261/318) of respondents reported that their schools were responsible for supplying soap from their own budgets. More than 90% (256/284) of respondents reported that their schools regularly cleaned areas and items that are more likely to have frequent hand contact, and more than 65% (173/284) of respondents reported that their schools cleaned the areas at least once a day. Frequently cleaning such areas in the school was more likely to be reported in schools with an FRT than in schools without (85% vs. 74%, p = 0.016) (Table 2).

### Perceived Severity of pH1N1

During the spring 2009 term, 56% (180/323) of respondents reported that their schools actively screened students and staff for signs and symptoms of flu. Five percent (17/323) of respondents reported that their school closed because of concerns about pH1N1, and 38% (122/321) of respondents reported that pH1N1 caused a substantial amount of illness in their school during the spring of 2009. K-5 and K-8 grade schools were more likely than 9–12 grade schools to report a substantial amount of illness in the spring of 2009. Schools located in Queens were the most likely to report substantial spring 2009 ILI. Schools reporting substantial illness in the spring had a slightly lower percentage of black or African American students and a higher percentage of Asian or Native Hawaiian/other Pacific Islander students (Table 3). Overall, NPI implementation did not differ by school according to reported ILI levels, except that schools that reported substantial spring ILI were also more likely to have reported an FRT (p = 0.017), tissue reported available in classrooms (p = 0.019), and hand sanitizer reported available in classrooms (p = 0.024), compared with schools without reported substantial spring ILI (Table 3).

**Table 3:** Characteristics of NYC public schools reporting and not reporting substantial ILI in spring 2009.

Characteristic	Reporting substantial Spring 2009 ILI		
	Yes (%yes)	No (%no)	P-value^a^
**Instructional level**	***n = 122***	***n = 199***	**0.002** b
Elementary schools (K-5)	66 (54%)	100 (50%)	
K-8 Schools	30 (25%)	32 (16%)	
K-12	4 (3%)	4 (2%)	
Middle schools (6–8)	17 (14%)	20 (10%)	
Middle/High (6–12)	1 (1%)	6 (3%)	
High schools	4 (3%)	27 (14%)	
Other	0	10 (5%)	
**Borough**	***n = 122***	***n = 199***	**0.002** b
Bronx	12 (10%)	30 (15%)	
Brooklyn	23 (19%)	61 (31%)	
Manhattan	19 (16%)	33 (17%)	
Queens	62 (51%)	58 (29%)	
Staten Island	6 (5%)	17 (9%)	
**Title I eligibility** *			
**Title I**	***n = 122***	***n = 189***	0.474^b^
School wide program	106 (87%)	160 (85%)	
No school wide program	16 (13%)	29 (15%)	
**Poverty rate** †	***n = 122***	***n = 189***	0.529^b^
0–25%	16 (13%)	23 (12%)	
25–50%	13 (11%)	23 (12%)	
50–75%	53 (43%)	68 (36%)	
75–100%	40 (33%)	75 (40%)	
**School size**	**n = 122**	**n = 189**	0.594^b^
<500 students	42 (34%)	68 (36%)	
500–1000 students	58 (48%)	95 (50%)	
>1000 students	22 (18%)	26 (14%)	
**Ethnicity** ¶	**n = 122%mean (95% CI)**	**n = 199%mean (95% CI)**	
American Indian or Alaska native	1% (0.39–0.62)	<1% (0.42–0.54)	0.655§
Black or African American	29% (23.5–33.7)	35% (30.7–39.5)	0.058§
Hispanic or Latino	34% (30.3–38.7)	37% (33.3–40.6)	0.382§
Asian or Native Hawaiian/Other Pacific Islander	**19% (15.4–23.3)**	**12% (9.4–14.2)**	**0.002** §
White	16% (12.7–20.3)	15% (11.8–18.1)	0.534§
**Responses (** ***number of survey respondents*** **)**			
Formation of a Flu Response Team (n = 321)	**95/122 (78%)**	**126/199 (63%)**	**0.017**
Designated or had a room used exclusively for separating ill students (holding room) (n = 318)	77/121 (64%)	131/197 (67%)	0.213
Educated or encouraged students to use respiratory etiquette (n = 321)	120/122 (98%)	197/199 (99%)	0.436
Educated or encouraged students to use proper hand hygiene (n = 321)	121/122 (99%)	197/199 (99%)	0.692
More than 75% of teachers taught curriculum on hand hygiene and respiratory etiquette (n = 320)			
During September 2009	78/122 (64%)	104/198 (53%)	0.384
During October 2009	70/122 (57%)	103/198 (52%)	0.27
During November 2009	63/122 (52%)	88/198 (44%)	0.62
During December 2009	63/122 (52%)	88/198 (44%)	0.582
Tissue was made available in these location (s) in school (n = 320)			
Classrooms	**115/122 (94%)**	**170/198 (86%)**	**0.019**
Bathrooms	66/122 (54%)	100/198 (51%)	0.532
Hallways	1/122 (1%)	5/198 (3%)	0.275
Admin offices	107/122 (88%)	164/198(83%)	0.239
Lunch room	41/122 (34%)	58/198 (29%)	0.418
Hand sanitizer was made available in these locations in school (n = 320)			
Classrooms	**112/122 (92%)**	**164/198 (83%)**	**0.024**
Bathrooms	48/122(39%)	62/198(31%)	0.142
Hallways	13/122 (11%)	17/198(9%)	0.537
Admin offices	103/122 (84%)	159/198 (80%)	0.352
Lunchroom	52/122 (43%)	65/198 (33%)	0.077
Cover your cough posters were made available in these locations in school (n = 320)			
Classrooms	50/122 (41%)	91/198 (46%)	0.384
Bathrooms	52/122(43%)	92/198(46%)	0.502
Hallways	96/122 (79%)	151/198(76%)	0.616
Admin offices	57/122 (47%)	87/198 (44%)	0.627
Lunchroom	73/122 (60%)	114/198 (58%)	0.69
Frequently cleaned areas and items that are more likely to have frequent hand contact (n = 318)	103/121 (85%)	153/197 (78%)	0.069
Communicated with students about flu at least once using the following methods (n = 317)			
Letters or handouts	107/121 (88%)	167/196 (85%)	0.191
School assemblies	88/121 (73%)	119/196(61%)	0.089
E-mails	14/121 (12%)	26/196 (13%)	0.364
Social Media (Facebook, twitter, etc)	1/121 (1%)	4/196 (2%)	0.098
Internet	**34/121 (28%)**	**66/196 (34%)**	**0.038**

a Participating schools compared to nonparticipating schools.

b Chi-square test.

§ Satterthwaite T-test of Mean Difference, two-sided exact **Pr>|t|**.

* A federal program that provides financial assistance to Local Education Agencies and schools with high numbers or high percentages of poor children.

† The percentage of public schools where ≥75% of students are eligible for free or reduced price lunch.

¶ Proportion of each ethnic group in schools.

## Discussion

This paper describes schools' capacity to implement non-pharmaceutical interventions during the pandemic influenza (specifically pH1N1) among school-aged children by a large public school system in the United States. The NYCDOE is the largest system of public schools in the United States, serving about 1.1 million students in nearly 1,700 schools [28]. Our survey findings suggest that many public schools implemented many of the recommended NPIs by the NYC health and school officials. During the 2009 fall term, nearly all respondents reported teaching curriculum on proper hand hygiene and respiratory etiquette. Implementation of other NPIs was variable.

Planning for an influenza outbreak in public schools was one of the hallmarks of DOHMH mitigation efforts against pH1N1. The capacity of OSH in getting schools to implement the guidelines cannot be fully assessed based on this evaluation, but evidently a high percentage of the survey participants implemented recommendations for planning for an influenza outbreak. Two key recommendations in the DOHMH planning guidelines are formation of a Flu Response Team as a part of school emergency preparedness plan, composed of school administrators, health officials, and parents, and designation of a holding room within a school to be used exclusively for separating persons with ILI symptoms. The majority of respondents, but not all, had a Flu Response Team and a holding room. The barriers to adoption of these key recommendations are unclear, but schools without an FRT were more likely to serve older students and had a smaller percentage of white students than those with an FRT. Moreover, schools with an FRT were more likely to implement more aspects of the mitigation guidelines, including isolating students with ILI symptoms, than were schools without an FRT, highlighting the importance of planning as a significant step in implementation of the mitigation guidelines. It is possible that many schools that did not respond to the survey did not have an FRT as part of their required emergency preparedness plans. It is also possible that these schools were less likely to implement the mitigation guidelines. However, other studies that looked at the use of NPIs to limit the spread of pH1N1 in schools revealed that many schools and universities in other parts of the United States adopted most CDC-recommended NPIs but compliance with certain NPIs, especially isolating students with ILI symptoms, was low [29], [30], [31]. These findings underscore the need to provide feasible recommendations that incorporate individual school needs and to allocate resources to address barriers to planning for influenza and other respiratory disease outbreaks and adoption of mitigation guidelines. Barriers to planning for pH1N1 outbreak by individual schools should be identified and addressed to allow successful implementation of mitigation measures by schools during future outbreaks.

Schools that reported substantial spring 2009 ILI were more likely to also report implementing the two key recommendations about planning for pH1N1. Many of these schools may have implemented the two key recommendations on planning before their schools experienced any significant pH1N1 disease; however some of these schools may have implemented the two recommendations in reaction to pH1N1 after experiencing outbreaks during the spring 2009. Guidance is needed to effectively integrate preparedness into everyday activities of schools to improve school responses during future influenza outbreaks.

Although fewer than half of respondents reported a substantial amount of illness in their school during the 2009 spring term, the majority of respondents reported that their interventions during the 2009 fall term made a difference in preventing influenza in their schools. This perception might have been influenced by the fact that there was little disease from pH1N1 in NYC public schools during fall term 2009 compared with the spring term 2009 [13]. The majority of respondents perceived NPIs as being effective in preventing influenza transmission. This perception could be due in part to the effort made by local and national public health authorities to promote school mitigation measures. This point is also highlighted by the fact that faculty and staff used their own funds to purchase hand sanitizer and tissue for their students in more than half of schools in the survey.

Schools reported communicating with students and parents using different methods and languages, but it is unknown how many students, parents or guardians received the communications from schools. In NYC, schools reported using mostly traditional methods of communication, including school wide handouts, letters, and student assemblies. Electronic communication methods such as e-mailing, mass texting, World Wide Web, social media were seldom used. Expanding the use of electronic methods of information sharing may enhance communication with students and parents during future influenza outbreaks. CDC is currently conducting a study to evaluate communication between schools and parents during the pH1N1 outbreak in Michigan.

This online survey had a number of limitations. Although the voluntary survey was e-mailed to all NYC public school principals by school officials, less than one-quarter of schools accessed the online survey, nearly all of whom responded to the survey. This suggests that the online format of administering the survey may have impacted the rate of participation by schools. In addition, there were only 2 weeks available to administer the survey and it was not possible to determine if each school received the study information sent via an email by NYCDOE using a general electronic mailing list. In similar studies done in the states of Georgia, Pennsylvania and Michigan, where a combination of web- and paper-based surveys were used, the responses rates ranged from 35% to 44% [29]. In NYC, the response rate varied by the grade levels of the school and the school size. For example, schools with lower grade levels (K-5) were more likely to respond than schools with higher grade levels (9–12 grades). This variation could be due in part to the fact that young children, who would typically be in grades K-5, were disproportionately affected by pH1N1 during spring 2009. The survey was completed by one or a few people familiar with administrative and health services at the school, but responses may not accurately reflect the plans, actions, and experiences of all school officials. Because answering some questions in the survey required recalling information, this evaluation may be subject to recall and social-desirability bias. Additionally, respondents did not provide data for all survey questions. Moreover, most respondents were from Queens, a borough that experienced substantial pH1N1 activity in the spring of 2009 and hence, the findings from our sample may not be generalizable to the entire public school system in NYC. However, because the school system in NYC is fairly centralized and the resources needed to implement NPIs were provided by the NYC school and public health officials, it is less likely that the findings from this study would have been significantly different if more schools from other boroughs participated in the survey. Measuring the effects of individual strategies used by OSH to disseminate pH1N1 mitigation guidelines to schools is beyond the scope of this evaluation and should be assessed to help the OSH and the school system determine the most effective strategies for disseminating future mitigation guidelines. Finally, we were unable to assess the impact of the recommended NPIs on pH1N1 transmission and ILI.

## Conclusions

The New York City health and school officials were able to implement a mitigation plan in NYC public schools during the 2009 academic year. The majority of the schools that participated in the survey received the DOHMH guidelines and adopted many of the recommendations. Schools differed in the recommendations they adopted. A better understanding of the issues and perceived challenges for schools in deciding which recommendations to implement would be beneficial for future pandemic preparedness planning.

The findings from our evaluation highlight the feasibility of implementing an NPI program in a large school system to mitigate the effects of an influenza outbreak, but also demonstrate the potential need for additional resources in some schools to increase capacity and adherence to all recommendations. The results provide insight into the implementation of local and national guidelines by individual schools during pH1N1 outbreak. Further research is needed to assess barriers to implementation of local and national guidelines and to develop best practices in preparation for future influenza outbreaks.
